# Cytokine Responses to Adenovirus and Adenovirus Vectors

**DOI:** 10.3390/v14050888

**Published:** 2022-04-24

**Authors:** Svetlana Atasheva, Dmitry M. Shayakhmetov

**Affiliations:** 1 Lowance Center for Human Immunology, Departments of Pediatrics and Medicine, Emory University School of Medicine, Atlanta, GA 30322, USA; 2 Emory Vaccine Center, Emory University School of Medicine, Atlanta, GA 30322, USA; 3 Discovery and Developmental Therapeutics Program, Winship Cancer Institute of Emory University, Atlanta, GA 30322, USA

**Keywords:** adenovirus, inflammation, cytokines, innate immunity, cytokine storm syndrome

## Abstract

The expression of cytokines and chemokines in response to adenovirus infection is tightly regulated by the innate immune system. Cytokine-mediated toxicity and cytokine storm are known clinical phenomena observed following naturally disseminated adenovirus infection in immunocompromised hosts as well as when extremely high doses of adenovirus vectors are injected intravenously. This dose-dependent, cytokine-mediated toxicity compromises the safety of adenovirus-based vectors and represents a critical problem, limiting their utility for gene therapy applications and the therapy of disseminated cancer, where intravenous injection of adenovirus vectors may provide therapeutic benefits. The mechanisms triggering severe cytokine response are not sufficiently understood, prompting efforts to further investigate this phenomenon, especially in clinically relevant settings. In this review, we summarize the current knowledge on cytokine and chemokine activation in response to adenovirus- and adenovirus-based vectors and discuss the underlying mechanisms that may trigger acute cytokine storm syndrome. First, we review profiles of cytokines and chemokines that are activated in response to adenovirus infection initiated via different routes. Second, we discuss the molecular mechanisms that lead to cytokine and chemokine transcriptional activation. We further highlight how immune cell types in different organs contribute to synthesis and systemic release of cytokines and chemokines in response to adenovirus sensing. Finally, we review host factors that can limit cytokine and chemokine expression and discuss currently available and potential future interventional approaches that allow for the mitigation of the severity of the cytokine storm syndrome. Effective cytokine-targeted interventional approaches may improve the safety of systemic adenovirus delivery and thus broaden the potential clinical utility of adenovirus-based therapeutic vectors.

## 1. Introduction

The innate immune system is the first line of defense against invading pathogens through recognition of conserved pathogen-associated molecular patterns (PAMPs). PAMPs frequently represent indispensable and integral parts of invading microorganisms [1] and include cell-wall components of bacteria or fungi, dsRNA molecules of viral genomes, and highly repetitive symmetry structures that are absent in the mammalian host [2,3]. The presence of PAMPs warrants swift activation of the immune system to initiate protective host immune response. The innate immune system is comprised of a network of tissue-resident and circulatory cells equipped with molecular machinery capable of recognizing PAMPs of invading microorganisms. Following the recognition of a pathogen, the innate immune system initiates the activation and release of cytokines and chemokines to alert surrounding tissues and the whole organism of the presence of a pathogen [4]. Cytokines and chemokines function as signal molecules that activate multiple cell-intrinsic and cell-extrinsic inflammatory and immune defense programs that ultimately synergize to clear the infection and protect the host [5]. Cytokines are potent mediators of cell–cell communication that can support cell survival or initiate cell death [5,6]. Chemokines attract various inflammatory immune cells [7] that can directly kill virus-infected cells and promote further release of inflammatory cytokines through feed-forward signaling amplification loops. While some cytokines and chemokines have a narrow spectrum of action, others have pleiotropic effects and are required for homeostatic functions, proper development, and functional maturation of various cell types [8]. Given their critical role and potency in triggering biological responses, the transcription, translation, and release of cytokines and chemokines are tightly regulated to avoid exuberant acute or latent collateral tissue damage and cytokine storm syndrome, which can lead to death [9]. Not surprisingly, de-regulated and exuberant cytokine and chemokine production and systemic release lead to persistent and frequently life-threatening pathologies.

## 2. Cytokine Responses to Adenovirus in Different Biological Contexts

Adenoviruses and adenovirus-based vectors are widely used in basic research, translational studies, and in clinic alike. In this review, we discuss cytokine responses to adenoviruses in the context of several therapeutic applications, including those where adenoviruses are used as vectors for vaccination (adenovirus vectors expressing an antigen for eliciting an antigen-specific immune response), for the therapy of cancer (oncolytic viruses), or for treating genetic diseases (gene therapy). However, these definitions and terminologies are rather fluid and interchangeable, as any expression of a gene of interest from the engineered virus may also be considered a gene therapy.

**Figure 1:**
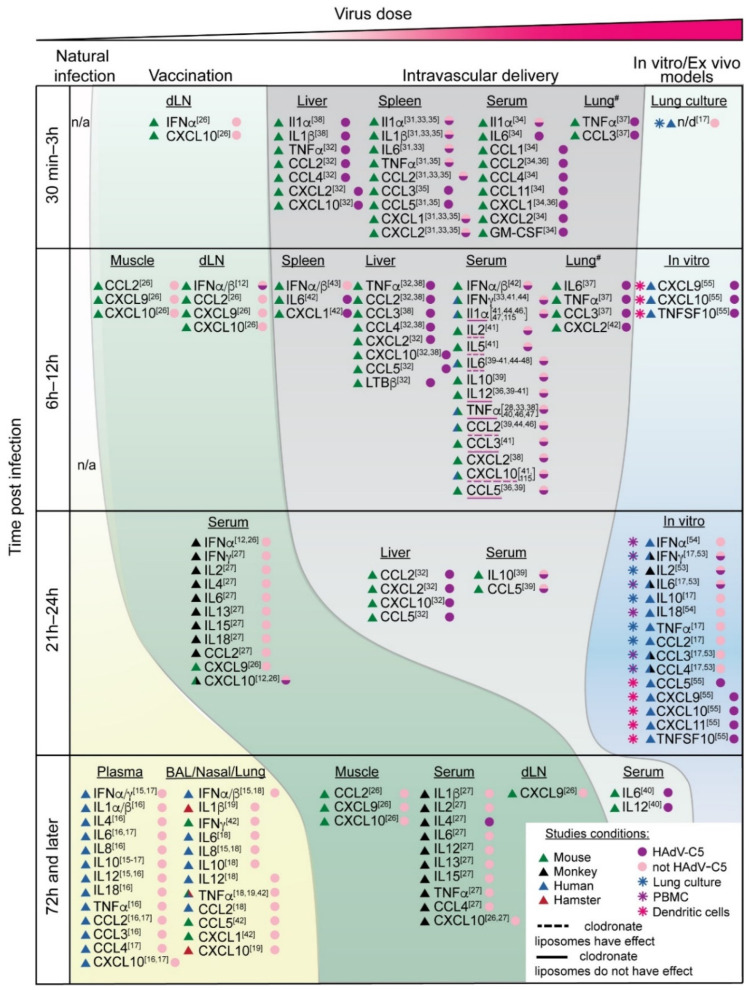
Cytokine responses to adenovirus in different biological contexts. The four areas are shaded in different colors, indicating adenovirus infection in various routes. The areas are positioned from left to right according to the virus dose received during infection. Vertically, the table is divided into four time periods from the earliest to the later times post infection. The deepest hues of the gradient colors show the peak times of cytokine response. The triangles indicate the species examined in the studies, and the color of the circles shows which adenovirus species were used. dLN—draining lymph node; BAL—bronchoalveolar lavage; n/a—data are not available; n/d—non detected; #—in this study, the virus was delivered intratracheally. The data for individual cytokines and chemokines, reported in references from [12,13,14,15,16,17,18,19,20,21,22,23,24,25,26,27,28,29,30,31,32,33,34,35,36,37,38,39,40,41,42,43,44,45,46,47,48,49,50,51,52,53,54,55,56,57,58,59,60,61,62,63,64,65,66,67,68,69,70,71,72,73,74,75,76,77,78,79,80,81,82,83,84,85,86,87,88,89,90,91,92,93,94,95,96,97,98,99,100,101,102,103,104,105,106,107,108,109,110,111,112,113,114,115], are shown with superscript numbers.

Although the exposure of immune cells to adenovirus triggers a rather stereotypic pro-inflammatory response with a defined set of cytokines and chemokines becoming activated shortly after detection of adenovirus particles, the magnitude and the spectrum of cytokine and chemokine activation varies greatly and depends on the initial virus dose that enters the host (Figure 1). Natural infection with adenovirus is believed to be initiated by a very small number of infectious viral particles, and thus, the initial recognition by the immune system leads to only subclinical and/or locoregional immune responses. In contrast, upon vaccination or systemic treatments with adenovirus-based vectors, the amounts of virus particles administered into the body are orders of magnitude higher than naturally occurring infection. Specifically, clinical trials have demonstrated that adenovirus-based vaccines are highly effective and generate protective immunity when they are administered intramuscularly at doses that range from 10^9^ to 10^11^ adenovirus particles [10,11]. At the vaccine injection site, the virus infects muscle cells in addition to being sequestered by the antigen-presenting cells, which transfer the virus to the draining lymph nodes, leading to the generation of both antigen- and virus-specific humoral and cellular immune responses through mechanisms of adaptive immunity [12]. Furthermore, systemic intravenous injection of adenovirus vectors for the therapy of metastatic cancer or genetic diseases requires even higher amounts of therapeutic virus, and the administered virus amounts can reach up to 3.8 × 10^13^ viral particles per single injection dose [13].

### 2.1. Natural Infection

Most human adenoviruses cause self-limiting illnesses of the conjunctiva, respir-atory tract, or gastrointestinal tract that resolve on their own within 7–10 days, with-out a need for hospitalization or medical intervention [14]. Therefore, data on the cy-tokine response in humans to the most common adenoviruses that cause respiratory illness, particularly species C adenoviruses (HAdV-C2 and HAdV-C5), are not availa-ble. However, infections with some human species B adenoviruses (e.g., HAdV-B3 and HAdV-B7) can lead to severe respiratory illness and manifest as pneumonia and acute respiratory distress syndrome (ARDS) [14]. Recently, the levels of cytokines and chemokines in plasma and bronchoalveolar lavage fluid (BALF) in immunocompetent adult and pediatric patient cohorts with pneumonia and ARDS caused by HAdV-B3 and HAdV-B7 infections were reported [15,16,17,18]. The amounts and the profiles of cyto-kines were determined in these patient cohorts were cytokines with potent pro-inflammatory properties, including IL-1α, IL-1β, IL-6, IL-8, IL-12, IFN-γ, IFN-α2, TNF-α, and chemokines CCL2, CCL3, and CXCL10. Interestingly, the same spectrum of cytokines was observed in BALF, nasal samples, and serum, suggesting that the cytokine response to adenovirus infection is stereotypic and is both local and systemic [15]. Moreover, clinical observations suggest that more severe disease is associated with prolonged expression and higher release of pro-inflammatory cytokines [16]. In patients with severe pneumonia and ARDS, these cytokines lingered for more than two weeks, while in milder cases, they subsided by 14 days after symptom onset [16].

Human adenoviruses exhibit species-restricted phenotypes, making studying disease progression in animal models particularly problematic. However, studies of HAdV-B14p1 in the Syrian hamster model have shed some light on virus-mediated immune- and histopathologies [19]. HAdV-B14p1 is a species B adenovirus (similar to HAdV-B3 and HAdV-B7) implicated in severe pneumonia and ARDS in humans [14]. The infection of Syrian hamsters with HAdV-B14p1 led to a more severe ARDS-like lung pathology compared to infection with the parental HAdV-B14 virus [19]. In addition to more extensive lung tissue damage, lung immune-pathology in this model included the high expression of pro-inflammatory cytokines IL-1β and TNF-α and chemokines CCL3 and CXCL10 [19]. HAdV-B14p1-infected cells had lower expression of the viral protein E1B 20K, an inhibitor of the host immune response [20], suggesting that the failure to inhibit pro-inflammatory cytokine production may be responsible for, and lead to, exuberant host pro-inflammatory immune activation and tissue damage [19].

Experimental infection with non-human adenoviruses, for instance, mouse adenovirus 1 (MAV1), triggers the activation of a similar set of cytokines during respiratory disease in mice comparable to human adenovirus infection in humans. The amounts of cytokines IFN-γ and TNF-α, as well as chemokines CXCL1, CCL2, and CCL5, were significantly elevated in the BALF of MAV1-infected mice on day seven post-infection [21]. A fowl adenovirus, known to cause severe disease in poultry populations, induced elevated mRNA transcription for the same spectrum of pro-inflammatory cytokines detected following mouse and human adenovirus infection (IL-1β, IL-6, IL-8, IFN-α, IFN-β, IFN-γ) [22]. Interestingly, an intramuscular route of infection was associated with higher cytokine expression in the spleen and led to more severe symptoms relative to an oral route of infection [22].

In summary, the cytokines and chemokines released in response to adenovirus infection are pro-inflammatory in nature and include cytokines IL-1α, IL-1β, IL-6, IL-8, IFN-γ, IFN-α, and TNF-α and chemokines CCL2, CCL3, and CXCL10. This set of cytokines and chemokines is predominant across species ranging from humans to birds. These pro-inflammatory cytokines are thought to be essential for mounting a protective anti-viral immune response. However, their continued activation may trigger a feed-forward “amplification loop” of pro-inflammatory signaling, when the initial expression of pro-inflammatory cytokines attracts the cells of hematopoietic origin to the site of infection. This in turn produces more and a wider spectrum of pro-inflammatory cytokines, attracting even more innate immune cells of hematopoietic origin that locally release proteins with potent anti-microbial and anti-viral properties, leading to collateral tissue damage and associated pathologies [23]. Taken together, the severity of the natural adenoviral disease appears to directly correlate with the amounts and the duration of the production of cytokines and chemokines at the site of the infection.

### 2.2. Vaccination

Adenovirus-based vectors are highly effective vaccine platforms. Under the severe pressure of the rapid and global spread of SARS-CoV-2 coronavirus, the causative agent of COVID-19 respiratory disease, adenovirus-based vaccines against SARS-CoV-2 were developed and deployed in the span of just a few months by several counties. To date, adenovirus-vectored vaccines against SARS-CoV-2 based on HAdV-D26 (Janssen Vaccines, Johnson & Johnson, New Brunswick, New Jersey, United States) [11], ChAdOx1 (Astra-Zeneca, Oxford, United Kingdom) [24], HAdV-D26/HAdV-C5 (Sputnik V vaccine Gamaleya Research Institute of Epidemiology and Microbiology, Moscow, Russia) [10], and HAdV-C5 (Ad5-nCoV, Sinopharm and CanSinoBIO, Tianjin, China) [25] have been administered to billions of people around the world and have undoubtedly saved millions of lives. The effectiveness of adenoviruses as vaccine vectors is based on their ability to elicit potent CD8+ T-cell responses and robust humoral immunity [12]. However, despite billions of people receiving the adenovirus-based vaccines, data on the profile of cytokines released in response to vaccinations are limited. Therefore, we will review the available data for vaccination studies performed in pre-clinical mouse and non-human primate models (Figure 1, Vaccination).

In mice, after intramuscular vaccine administration, IFN-α and CXCL10 are detected three hours post vector injection in the local draining lymph nodes but not in the muscle tissue [26]. Six hours post-vaccination, these proteins can be detected not only in the draining lymph nodes but also in muscle tissue surrounding the injection site [12,26]. In the blood, elevated amounts of IFN-γ, IL-2, IL-6, IL-12, IL-15, and IL-8 cytokines, in addition to chemokines CXCL9 and CXCL10, are observed only at 24 h post-vaccine administration [12,26,27]. These same cytokines and chemokines are elevated in the plasma of monkeys at seven days post-vaccination [26,27].

It is noteworthy that vaccinations in animal models with non-HAdV-C5-based vectors led to the production of higher amounts and prolonged expression of pro-inflammatory cytokines and chemokines in comparison to inoculation with HAdV-C5 vectors [12,27]. This observation is reminiscent of a more potent pro-inflammatory responses to non-HAdv-C5 adenovirus serotypes upon natural infection. While HAdV-C5 causes self-limiting common cold illness in immunocompetent hosts, the non-HAdV-C5 adenovirus species cause more severe respiratory disease and activate robust pro-inflammatory cytokine production that may last longer than two weeks [16].

### 2.3. Intravascular Administration

In addition to being a highly effective vaccine platform, adenovirus vectors are a very promising gene delivery platform for the therapy of genetic diseases and oncolytic platform for therapy of localized and disseminated cancers. For many of these applications to be effective, the virus must be injected intravascularly (i.v.). Similar to other gene delivery platforms, after i.v. administration, the majority of the injected dose of adenovirus vector is sequestered within the reticulo-endothelial system of the liver and spleen due to the specialized tissue architecture and the abundance of innate phagocytic cells in these organs that sequester pathogens from the blood [28,29]. In the settings requiring i.v. administration of the therapeutic virus, the virus dose that enters the bloodstream over a very short period of time is exceptionally high, reaching up to 3.8 × 10^13^ viral particles [13]. It is critical to note that despite adenovirus vectors for cancer and gene therapy applications being engineered to be profoundly attenuated and to have very limited or no capacity for replication in normal tissues, the immune system still recognizes therapeutic vectors as genuine viral pathogens due to the PAMP moieties present in the adenovirus capsid or through sensing virus entry into cells (see below). Therefore, upon injection of extremely high amounts of virus particles in the bloodstream, the immune system promptly recognizes the presence of the virus and activates innate immune defensive mechanisms that trigger the production of inflammatory cytokines and chemokines. The timing of the cytokine response after i.v. injection strongly supports this model (Figure 1). Whereas after natural infection the significant amounts of cytokines were only detected at 7 days post-infection, after vaccination, cytokines in the serum appear within 24 h. In the case of i.v. injection of the adenovirus to mice, elevated transcription of the pro-inflammatory cytokine IL-1α can be detected as early as 10 min post-virus injection [30]. At one-hour post-virus injection, elevated levels of the “early response” pro-inflammatory cytokines IL-1, IL-6, IL-8, TNF-α and chemokines CCL2, CCL3, CCL4, CXCL1, CXCL2, CXCL9, and CXCL10 can be detected in the liver, spleen, blood, and lungs [30,31,32,33,34,35,36,37,38] (Figure 1, Intravascular delivery). Peak concentrations of the major early response pro-inflammatory cytokine IL-6 in the blood occurs at six hours post-i.v. virus injection in mice [32,33,37,38,39,40,41,42,43]. Almost none of the early response cytokines continue to be present in the blood at 24 h post-i.v. virus injection.

In humans, the elevation of pro-inflammatory cytokines in the blood after i.v. injection of adenovirus vectors is dose-dependent and occurs with kinetics similar to those observed in mice. IL-1 is one of the first pro-inflammatory cytokines that appears in the blood at three hours post-virus administration; however, by 18 h post-virus injection, the amount of IL-1 in the blood returns to baseline levels [44]. Blood levels of other early response cytokines, namely, IL-6, TNF-α, and IFN-γ and the CCL2 chemokine, are also increased and peak at 6 h after i.v. injection of adenovirus vectors [44,45,46,47,48]. By 24 h post-injection, the amounts of these cytokines in the blood subside to the baseline levels [44,45,46]. It is noteworthy that compared to HAdV-C5-based vectors, i.v. administration of the non-HAdV-C5-based vectors induced more potent elevation of IL-6 in the blood, albeit early cytokine responses in humans were measured for only one non-HAdV-C5-based adenovirus vector, namely, a group B adenovirus HAdv-B11 [46,48].

In contrast to i.v. administration of adenovirus vectors in humans, clinical trials employing intra-tumoral virus injection showed that IL-6 release in response to the first virus injection is not substantial and mostly confined to its normal ranges [49,50,51,52]. However, repeated intra-tumoral injection may lead to elevated IL-6 concentrations in the blood, though at much lower levels than after i.v. virus injection [49,50].

Taken together, regardless of the delivery route, following detection of the adenovirus or adenovirus vectors by the innate immune system, the transcription of the same set of the early response pro-inflammatory cytokines, namely, IL-1, IL-6, IL-8, TNF-α, and the CCL2, CCL3, CCL4, CXCL1, CXCL2, CXCL9, and CXCL10 chemokines becomes activated, leading to their production and release. Moreover, the cytokine profiles produced in response to adenovirus in the in vitro infection models of human or monkey PBMC, lung cultures, or blood-derived dendritic cells are also similar to those detected after vaccination, natural infection, or i.v. administration of adenovirus vector (Figure 1, In vitro models) [17,53,54,55]. The stereotypic nature of the innate immune response to adenovirus in diverse biological contexts strongly suggest that the activation of pro-inflammatory cytokines and chemokines in response to adenovirus is driven in different systems by the same molecular mechanisms.

## 3. Molecular Mechanisms Implicated in Activation of Cytokines and Chemokines in Response to Adenovirus

**Figure 2:**
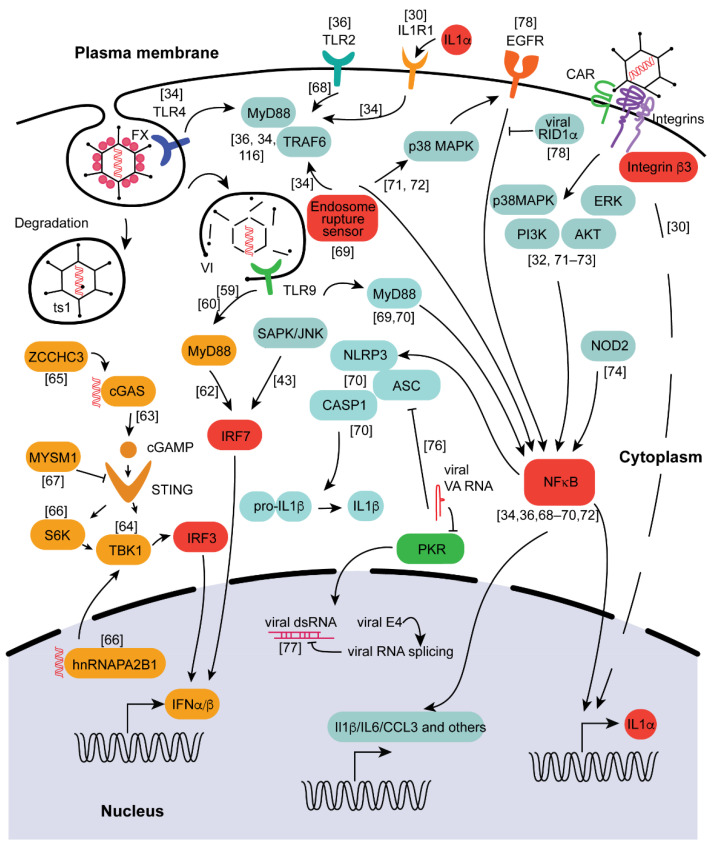
Molecular mechanisms triggering cytokine activation in response to adenovirus infection. Several main events trigger cytokine and chemokine activation during adenovirus infection. The hubs that play the major roles are shown in red. The orange molecules are associated with IRF3-dependent transcription. The teal color indicates the factors that signal through the NF-κB transcription hub. Integrin β3 activation of IL-1α and IL-1α-dependent augmentation of cytokine production are other main events leading to cytokine expression after adenovirus infection. The data for individual proteins, reported in references from [30,31,32,33,34,35,36,37,38,39,40,41,42,43,44,45,46,47,48,49,50,51,52,53,54,55,56,57,58,59,60,61,62,63,64,65,66,67,68,69,70,71,72,73,74,75,76,77,78,79,80,81,82,83,84,85,86,87,88,89,90,91,92,93,94,95,96,97,98,99,100,101,102,103,104,105,106,107,108,109,110,111,112,113,114,115,116], are shown with superscript numbers.

The innate immune system senses the presence and the entry of pathogens into cells through an array of cell-surface-localized and cytosolic receptors that recognize pathogen-specific PAMPs as well as pathogen-induced perturbations of cellular homeostatic functions. Sensing of the pathogen through this network of receptors triggers downstream signal transduction pathways that activate the expression of large sets of genes, many of which are directly involved in enabling host defense functions, including transcriptional activation of genes encoding pro-inflammatory cytokines and chemokines. In the case of adenoviruses, the transcription of cytokine and chemokine genes following detection of adenovirus entry into cells is driven by the IRF3, IRF7, and the NF-κB families of transcription factors [56]. The specific signal transduction pathways that were implicated in sensing adenovirus and that lead to activation of IRF3, IRF7, and NF-kB transcription factors are shown in Figure 2. Whereas the genes playing the key role in activating host antiviral responses, activated by IRF3 and IRF7, are genes encoding type I interferons, transcription factor NF-κB activates the expression of many genes encoding cytokines and chemokines, including IL-1β, IL-6, IL-8, IL-12, IL-18, CCL2, CCL3, CCL4, CXCL1, CXCL2, and CXCL10 [57]. In addition, some chemokines, such as CXCL10, are synergistically activated by both NF-κB- and IFN-I/STAT1-mediated signaling [58].

The transcription of the type I interferon genes can be activated by three different pathways depending on the cell type detecting the adenovirus. Virus disassembly in the endosomes and the exposure of viral DNA in plasmacytoid dendritic cells trigger the activation of the TLR9/MyD88 axis [59,60], leading to the IRF7-dependent transcription of IFN-I genes [61]. Alternatively, the detection of the viral DNA in the cytoplasm leads to the TLR-independent activation of type I IFN through cGAS/STING/TBK1/IRF3 signaling pathway [59,62,63,64]. Additionally, in mouse splenic myeloid dendritic cells, IFN transcription is independent of TLRs or IRF3 signaling but dependent on SAPK/JNK and IRF7 activation [43]. 

Several additional players that potentiate IFN production in response to adenovirus infection were recently identified. In the cytosol, cGAS that recognizes adenoviral dsDNA genomes cooperates with ZCCHC3 to allow for the highly efficient production of cGAMP, with subsequent activation of STING/IRF3-dependent transcription of IFN-I [65]. In the nucleus, protein hnRNAPA2B1 binds to viral DNA, leading to increased phosphorylation of TBK1 [66], thus potentiating signaling that activates IRF3. There are also players that the adenovirus targets to suppress IFN production and signaling. For example, adenovirus infection leads to increased transcription of MYSM1, a repressor of STING signaling, thus enabling the virus to reduce IFN-I expression activated through the STING/TBK1/IRF3 pathway [67].

The NF-kB-dependent transcription of pro-inflammatory cytokines can be activated downstream of multiple sensors of the Toll-like receptor (TLR) family, including TLR2, TLR4, and TLR9 [34,36], which transduce sensory signals through the MyD88/TRAF6/NF-kB axis [34,36,68,69,70]. In addition, depending on the cell type and the identity of the upstream receptor that detects adenovirus entry into the cell, different cellular serine/threonine phosphokinases and members of the MAPK pathway (including p38MAPK, ERK1/2, PI3K, AKT) also play a role in the transcriptional activation of cytokines and chemokines [32,71,72,73]. Cells that express components of the NOD-like receptor family (NLR), including NOD2 and NLRP3, or a non-NLR cytosolic DNA sensor absent in melanoma 2 (AIM2); adaptor protein ASC; and caspase-1 can respond to adenovirus by activating the inflammasome pathway [70,74]. NLRP3/ASC/Caspase-1 or AIM2/ASC/Caspase-1 inflammasome activation results in caspase-1-mediated cleavage of the pro-form of IL-1β to its mature functionally active cytokine form and its systemic release [70], which can subsequently activate an array of cytokines and chemokines via IL-1RI and the feed-forward amplification loop of pro-inflammatory signaling.

Similar to other viruses, adenoviruses evolved mechanisms to suppress pathways activating IFN-I, aiding in the evasion of IFN-I-dependent effector mechanisms. Adenovirus-encoded, virus-associated RNAs, transcribed by RNA polymerase III (VA RNAs) shortly after adenovirus genome entry into the nucleus, are well-established and highly effective suppressors of PKR [75]. In addition to its canonical function of arresting cellular protein synthesis, PKR is also required for the phosphorylation of the inflammasome adaptor ASC and its oligomerization [76]. Specifically, adenovirus VA RNAs arrest ASC oligomerization, thus preventing NLRP3 inflammasome formation and inhibiting functional maturation of IL-1β and other cytokines whose functional maturation depends on caspase-1 processing [76]. Another strategy evolved by adenovirus to avoid IFN-I activation, and its antiviral effectors depends on the function of proteins encoded in the E4 region of the adenovirus genome. Specifically, the splicing of viral mRNAs produced following the bi-directional transcription of adenovirus proteins during virus replication is tightly regulated by adenoviral E4 proteins so that the formation of the IFN-I and PKR-activating dsRNA complexes of complementary mRNAs is avoided [77]. Yet another adenoviral protein, RID1α, encoded in the E3 region of the adenoviral genome, functions to avoid the NF-κB-dependent activation of cytokine and chemokine expression downstream of the EGFR signaling [78].

Strikingly, the analysis of pro-inflammatory responses to adenovirus has demonstrated that the transcriptional and/or functional activation of cytokines and chemokines strongly depends on the viruses’ ability to rupture cellular endosomes [34,69,71]. Adenovirus mutant *ts1*, which cannot escape cellular endosomal compartments through endosome rupture [79], has reduced the activation of cytokines and chemokines, compared to virus variants that can efficiently escape endosomal compartments after virus internalization into the cell [30,43,71]. IL-1α is one of the earliest pro-inflammatory cytokines transcriptionally activated in splenic MARCO+ and CD169+ marginal zone macrophages after i.v. administration of adenovirus vectors in mice [30]. Although IL-1α was transcriptionally activated within 10 min after administration for both the mutant *ts1* and unmodified HAdv-C5-based vector, the functional maturation of IL-1α and the activation of the IL-1α-IL-1RI-dependent pro-inflammatory signaling failed to occur after administration of the *ts1* mutant virus. In contrast, an HAdv-C5-based vector, capable of escaping endosomal compartments through endosome rupture, triggered not only early Il1a gene transcription but also functional maturation of IL-1α, manifested by its translocation into the nucleus and activation of an IL-1RI-dependent array of pro-inflammatory cytokines and chemokines, including IL-1β and IL-6, and the CCL2, CXCL1, and CXCL2 chemokines [30]. The extremely rapid activation of *Il1a* gene transcription after i.v. virus administration suggests that adenovirus sensing in macrophage cells occurs either at the plasma membrane or in early endosomal compartments. Indeed, the interaction of adenovirus penton RGD motif with β3 integrins displayed at the macrophage surface was necessary to trigger *Il1a* transcription and i.v. administration of an adenovirus mutant lacking the RGD amino acids in the penton protein or administration of the virus with unmodified capsid to mice deficient for β3 integrin expression failed to trigger *Il1a* transcriptional activation and initiation of the IL-1α-IL-1RI-dependent pro-inflammatory signaling cascade [30].

Taken together, these data strongly suggest that adenovirus is sensed by an array of innate immune receptors at every step of virus entry into the cell, including at the plasma membrane, endosomal compartments, cytosol, and even within the nucleus. Based on the dose-dependent nature of cytokine responses to adenovirus, it is plausible that during natural infection, which is initiated by a low dose of the virus, only some of the innate immune sensors become activated, leading to the production of low amounts and a limited number of inflammatory cytokines and chemokines. In contrast, upon i.v. administration of large amounts of adenovirus vectors, most, if not all, of the innate immune receptors become simultaneously engaged, triggering production of high amounts and a broad spectrum of inflammatory cytokines and chemokine. This highly potentiated cytokine and chemokine release by itself, as well as through the induction of a feed-forward amplification loop of the pro-inflammatory signaling, may culminate in the cytokine storm syndrome, severe systemic toxicity, and even death.

## 4. Cell Types That Produce Cytokines and Chemokines in Response to Adenovirus

As described above, transcriptional activation and production of cytokines and chemokines occurs following triggering of sensors of innate immunity that detect adenovirus particles at the cell surface or inside the cell. Not all cell types can recognize the invading microorganisms and mount activation of cytokine and chemokine genes. Tissue-resident macrophages in liver and spleen are highly efficient at removing blood-borne pathogens and are the first cell types that encounter the incoming virus after i.v. administration. The liver-resident macrophages, Kupffer cells, represent the largest pool of innate phagocytic cells in the body. They efficiently sequester blood-borne adenovirus particles via a variety of plasma membrane receptors, including complement receptor CRIg [80] and scavenger receptors SR1 [81,82] and CD36 [33]. Within 30 min of sequestering adenovirus particles from the blood, Kupffer cells activate the expression of IL-1α, IL-1β, TNF-α, CCL2, CCL3, CXCL2, and CXCL10 [38]. It is noteworthy that the expression of IL-6 was not detected in the liver, suggesting that the systemic IL-6 observed 6 h post-i.v. virus administration is driven not by Kupffer cells but by cell types residing in other organs, most notably the spleen. Moreover, within 1 h post-i.v. adenovirus administration, the majority of Kupffer cells that sequestered adenovirus particles undergo “defensive suicide”, a necrotic-type cell death associated with the loss of plasma membrane integrity, thus effectively reducing the amounts of circulating adenovirus in the blood and limiting virus spread to vital organs, most notably the liver [35,83]. Upon induction of a “defensive suicide” necrosis, Kupffer cells release their cytosolic contents into the surrounding milieu and simultaneously release the pro-inflammatory cytokines and other factors that activate both local and systemic inflammation [83]. It is plausible that Kupffer cells are primarily responsible for the early production and release of IL-1α and IL-1β into the circulation, as the plasma concentrations of these cytokines increase early and subside in the liver at later times when Kupffer cells disintegrate or are no longer physiologically active [38].

The splenic marginal zone CD169/MOMA+ and MARCO+ macrophages also efficiently sequester adenovirus from the bloodstream. Although the identity of the plasma membrane receptor(s) that mediate virus sequestration in marginal zone macrophages remains unknown, as discussed, the adenovirus penton RGD amino acids interact with β3 integrins on the surface of these phagocytic cells and trigger signaling, leading to the transcriptional and functional activation of IL-1α [30]. In turn, IL-1α triggers the activation of a variety of cytokines and chemokines, most notably IL-6, via its cognate receptor, IL-1RI [30]. Moreover, IL-1α is the principal mediator activating the CXCL1/2-CXCR2 chemokine signaling axis, leading to the influx and retention of neutrophils into the splenic marginal zone [31]. Local degranulation or release of cytotoxic factors by neutrophils leads to the elimination of adenovirus-containing macrophages from the spleen in 24 h, thus limiting systemic spread of adenovirus through the blood [31].

The key role of macrophages and other phagocytic cells in triggering cytokine production in response to adenovirus was confirmed in experiments using clodronate liposomes, which, upon i.v. injection, eliminate all phagocytic cells, including macrophages in the liver and spleen [84]. The elimination of all phagocytic cells prior to adenovirus injection in mice greatly reduces the amounts of IL-1, IL-2, IL-6, IL-12, and TNF-α cytokines and CCL2 and CCL3 chemokines in the blood after i.v. adenovirus administration (Figure 1, underlined), suggesting that phagocytic cells are the primary source of cytokines and chemokines released into the circulation in response to i.v. adenovirus injection [39,40,41].

The dendritic cells in the spleen are the principal source of type I IFNs, a set of the key anti-viral cytokines that trigger the upregulation of thousands of genes with various host defense and homeostatic functions [43,54,60]. In response to virus infection, many types of cells can produce IFN-α/β. However, dendritic cells generate orders of magnitude higher amounts of type I IFN than other cell types [59]. Dendritic cells can sequester the virus through cognate fiber receptors or DC-SIGN [85], and in addition to type I IFN production, they synthesize other pro-inflammatory cytokines. In vitro studies confirm that monocyte-derived dendritic cells can sequester adenovirus–antibody complexes, leading to the expression of type I IFNs and other inflammatory cytokines [86,87].

In an in vitro model of natural infection using human polarized mucosal epithelial cells and peripheral blood monocyte-derived macrophages, adenovirus infection and virus sequestration in macrophages activates IL-8 production [88]. The local production of IL-8 from macrophages initiates the re-localization of the adenovirus receptors from the basolateral to the apical surface of epithelial cells, making them susceptible to direct infection with the virus via the fiber protein, the principal cell attachment protein of the adenovirus [88].

Taken together, evidence suggests that tissue-resident innate phagocytic cells, primarily tissue-resident macrophages and dendritic cells, represent the principal compartment that sequesters adenovirus particles and triggers local and systemic cytokine and chemokine production. Consistent with their sentinel and scavenger functions, tissue-resident macrophages sequester large amounts of adenovirus particles from the blood and become promptly eliminated either through a “defensive suicide” necrosis, like the Kupffer cells in the liver, or through neutrophil-mediated cytotoxicity against marginal zone macrophages in the spleen. The production of pro-inflammatory cytokines and chemokines and elimination of virus-containing macrophages limit spread of the virus to vital organs and prompt initiation of adaptive immune responses to enable clearance of adenovirus in non-phagocytic cells throughout the body.

## 5. Factors Modulating Cytokine and Chemokine Production in Response to Adenovirus

**Figure 3:**
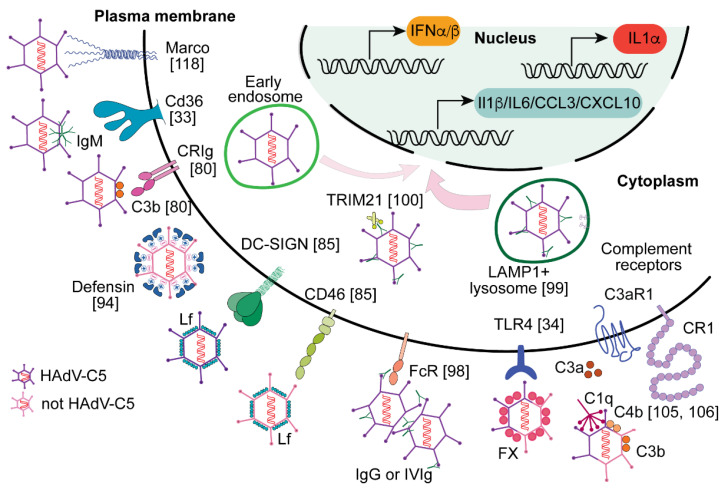
Factors enhancing cytokine and chemokine production in response to adenovirus infection. Different factors that bind to adenovirus particles can change the receptor selectivity and target the virus to macrophages and dendritic cells in the absence of the cognate virus receptors. The data for individual signaling molecules as reported in references from [33,34,35,36,37,38,39,40,41,42,43,44,45,46,47,48,49,50,51,52,53,54,55,56,57,58,59,60,61,62,63,64,65,66,67,68,69,70,71,72,73,74,75,76,77,78,79,80,81,82,83,84,85,86,87,88,89,90,91,92,93,94,95,96,97,98,99,100,101,102,103,104,105,106,107,108,109,110,111,112,113,114,115,116,117,118], are shown in parentheses adjacent to indicated proteins.

Upon entry into the cell, adenoviruses utilize a variety of primary attachment receptors, including CAR, CD46, DSG2, and sialic acid, as well as cellular integrins, which serve as co-receptors and mediate virus internalization into the cell [89,90,91,92]. Virus interaction with primary attachment receptors is mediated by the fiber protein, whereas virus interaction with cellular integrins is mediated by the penton. However, when virus particles enter the bloodstream, numerous factors in the blood recognize and bind to the virus. These blood factors effectively "tag" viral particles, and the virus–blood factor complexes interact with a different set of receptors and enter cells through mechanisms unavailable to the “naked” virus. Moreover, these cell surface receptors not only dictate which cell types the virus–blood factor complexes will enter but also how these cells may respond to the virus infection (Figure 3).

Upon searching for the natural mechanisms that may neutralize adenoviruses, it was found that human alpha defensin 5 (HD5), a small antimicrobial peptide, has potent inhibitory activity against species C adenovirus HAdV-C5 [93]. However, HD5 does not inhibit adenoviruses of species D and F, but rather augments the virus infection in vitro and increases adaptive responses to the virus-encoded transgene antigens in vivo [94]. Furthermore, pre-treatment of mice with HD5 prior to HAdV-D26 injection resulted in a higher cytokine response than to the virus alone. Mechanistically, more robust cytokine production in response to the virus–HD5 complexes is thought to be due to more efficient virus entry into dendritic cells in the presence of HD5. It is plausible that HD5 functions as a polycation, and upon binding to adenovirus, it shields the negatively charged amino acids present at the surface of the virion, thus reducing repulsion between the negatively charged virus surface and plasma membrane, leading to a more efficient infection of dendritic cells [94].

Lactoferrin (Lf) is another protein that enhances adenovirus entry into dendritic cells [95]. Specifically, it was shown that the bovine Lf enhances entry of human adenoviruses into dendritic cells more efficiently than human Lf, which can potentially be explained by the differential glycosylation patterns of these proteins [85]. Interestingly, the HAdV-C5-bLf complexes utilize the DC-SIGN receptor on dendritic cells, which is not a natural HAdV-C5 receptor, while HAdV-B35–bLf complexes require a cognate CD46 receptor for cell entry. Similar to the HD5-mediated enhancement of infection and cytokine production by the dendritic cells, the adenovirus–bLf complexes also trigger a more potent release of cytokines than the virus alone [85]. The higher efficacy of entry of adenovirus–bLf complexes into dendritic cells can potentially be explained by the engagement of alternate cell surface receptors, which are not utilized for cell entry by the virus without Lf.

Coagulation factor X (FX) binds to HAdV-C5 and other adenoviruses with low-nanomolar or even picomolar affinity [34,96]. FX binding to adenoviruses shields virus particles in the blood from other factors and enables virus escape from neutralization via natural IgM-dependent activation of the complement system [97]. Furthermore, in the mouse system, when HAdV-C5–FX complexes are sequestered by the splenic marginal zone macrophages, they activate a broader spectrum of inflammatory cytokines and chemokines, compared to a mutant virus that cannot bind to FX [34]. Importantly, the FX-mediated potentiation of cytokine activation was not observed in vitro when adenovirus–FX complexes were added to human dendritic cells differentiated from peripheral blood monocytes [98]. These data suggest that the sensing of adenovirus–FX complexes by the innate immune system may be species-specific or that potential differences in cell-intrinsic sensory mechanisms exist between tissue resident macrophages in vivo and in vitro differentiated dendritic cells.

Adenovirus-specific IgG antibodies can also potentiate virus entry into dendritic cells with subsequent activation of anti-viral and inflammatory cytokine production. In vitro, the pre-incubation of pooled human immunoglobulin (IVIg) with adenovirus leads to the formation of immunocomplexes consisting of adenovirus virions cross-linked by the antibodies [86]. Compared to the virus alone, these adenovirus immunocomplexes infect monocyte-derived human dendritic cells with higher efficacy [86]. Dendritic cells sequester these immunocomplexes via an array of Fc receptors, thus completely bypassing cognate adenovirus fiber receptors [99]. The pre-treatment of adenovirus with IVIg increased virus-encoded transgene expression in dendritic cells and potentiates cytokine production from these cells [86,87]. At least two mechanisms may contribute to the elevation of cytokine production by the dendritic cells in response to adenovirus immunocomplexes. One mechanism involves TRIM21, an intracellular Fc receptor that recognizes the adenovirus–antibody complexes in the cytosol, leading to elevated cytokine production from the cells [100]. The second mechanism that may contribute to potentiation of cytokine production in response to adenovirus immunocomplexes is aberrant intracellular trafficking to the late LAMP1-positive lysosomes. The release of adenovirus particles from the late lysosomes is associated with enhanced cytokine response compared to the virus particles that were released from the early endosomal compartments [53,99].

The elevated cytokine production in the presence of adenovirus-specific antibodies was confirmed in vivo in rodents and primates pre-immunized with HAdV-C5. In pre-immunized animals, the i.v. injection of a high dose of HAdV-C5 showed an augmented cytokine response, particularly at 6 h post-systemic virus injection [101,102]. However, pre-immunization almost completely prevented liver transduction and subsequent hepatotoxicity in animals [101,103]. Moreover, experiments in mice demonstrated that the enhanced cytokine production in response to adenovirus in pre-immunized animals depends in part on the functional complement system, particularly on the presence of complement component 3 (C3). C3 was implicated in potentiating neutrophil influx to the spleen after i.v. adenovirus administration [31], enhancing adenovirus-immunocomplex signaling [86], as well as in CRIg-mediated uptake of adenovirus particle by the Kupffer cells in the liver [79]. Furthermore, after i.v. administration, adenovirus triggers complement activation via both classical (antibody-dependent) and alternative pathways [104]. It remains unclear whether C3 triggers potentiated cytokine production via an anaphylatoxin C3a domain, which is released after proteolytic processing of C3, and can directly activate C3a receptors on macrophages and dendritic cells, or whether C3 is required to be covalently bound to the virus particles in the form of C3b and activate other complement receptors, CR1-CR4. It is certainly likely that upon complement activation, both products of proteolytic cleavage of C3 contribute to the inflammatory cytokine activation and release observed shortly after i.v. virus administration. Another complement component 4 (C4) blocks virus disassembly and thus effectively neutralizes the virus [105,106]. The specific role of C4-mediated adenovirus neutralization in cytokine production is currently unknown and requires further investigation. It is clear, however, that as a principal component of humoral innate immunity, the complement system becomes activated in response to systemic adenovirus administration. Complement activation contributes to direct virus neutralization and the activation of cytokine and chemokine production by the innate phagocytic cells, which enables virus elimination, promotes activation of adaptive immunity that leads to clearance from the body of virus-infected cells, and generates long lasting virus-specific immunity.

## 6. Management of Cytokine Responses to Therapeutic Adenovirus Vectors

The key driver for the extensive efforts to better understand acute inflammatory cytokine production and cytokine storm syndrome is a quest to find approaches to improve the safety of adenovirus-based vectors as therapeutic platforms for treating human diseases. The advantages of adenovirus vectors are many and include established, highly efficient, standardizable, and cost-effective manufacturing of therapeutic vector stocks, large payload capacity (allowing for delivery of therapeutic transgenes that cannot be accommodated by alternate vector systems), ability to target adenovirus vectors to specific cell types of interest through modification of virus capsid proteins, vector stability, the episomal nature of a double-stranded DNA viral genome that does not integrate into cellular chromosomes (thus minimizing risk of insertional mutagenesis) and well understood biology of virus reproduction cycle, allowing for the attenuation of virus virulence and improving the safety of this vector platform. However, as we discussed above, despite attenuation and even complete lack of capacity for replication due to numerous deletions in key regulatory genes in the adenovirus genome, therapeutic adenovirus vectors are still recognized by the innate immune system as genuine pathogens, and innate phagocytic cells mount a potent systemic inflammatory cytokine response that can lead to cytokine storm syndrome. This is particularly problematic when extremely high amounts of adenovirus particles are administered over a short period of time, especially via an i.v. route. The recognition of therapeutic vectors as genuine pathogens and subsequent activation of potent systemic inflammatory cytokine responses is not unique to adenovirus vectors and is also observed in clinical trials evaluating administration of high doses of therapeutic adeno-associated virus (AAV)-based vectors to patients with rare genetic diseases [107,108]. Furthermore, the early iterations of cell therapy based on CAR-T cells that revolutionized treatment of certain types of cancer triggered cytokine storm syndrome that required the development of mitigation strategies to improve the safety of this approach [109]. Specifically, the administration of cancer-specific CAR-T cells in combination with the anti-IL-6 receptor antibody, tocilizumab [110], or recombinant IL-1RI antagonist, Anakinra [111], allowed for a significant reduction in inflammatory cytokine production in response to CAR-T cells therapy. Because IL-1 and IL-6 were found to be some of the very first cytokines activated in response to i.v. administration of adenovirus vectors [44,47,48], it is plausible that interfering with IL-1 and/or IL-6 signaling with currently available FDA-approved drugs, may prove to be an effective strategy to suppress acute systemic cytokine activation after administration of therapeutic adenovirus vectors. Indeed, recent studies in non-human primates demonstrated that safety of systemic delivery of a high dose adenovirus vector could be improved when virus was administrated following a “cytokine prophylaxis”, pre-treatment of animals with tocilizumab, Anakinra, and a broadly suppressive dexamethasone [112]. While the suppression of cytokine production prior to administering adenovirus-based therapy may prove beneficial in the context of adenovirus-based gene therapy applications, this “cytokine prophylaxis” approach may not be useful when adenoviruses are administered to patients for therapy of cancer. It is broadly accepted that virus-based activation of pro-inflammatory type-I cytokines within tumor microenvironment and lymphoid tissues is essential for breaking the state of local and systemic immune tolerance to tumors and, thus, is necessary for efficient anti-tumor response after virotherapy. Future clinical trials are needed to show whether a tailored “cytokine prophylaxis” approach can be utilized for improving the safety of adenovirus vectors without deleterious effects on their efficacy as cancer therapeutics.

Another approach to improving the safety of adenovirus-based vectors is the modulation of virus–host interactions that lead to virus sequestration in immune phagocytic cells and activation of inflammatory cytokine production through the introduction of mutations into the virus capsid. Our group has recently reported that in HAdv-C5 capsid, natural IgM antibodies bind to the HVR1 region of the hexon protein. Accordingly, adenovirus vector with a mutated HVR1 hexon region failed to bind IgM in mouse and human sera and escaped sequestration in Kupffer cells after i.v. administration [33]. Furthermore, a substitution of RGD amino acids in the adenovirus penton protein for a laminin-derived peptide that cannot interact with macrophage β3 integrins resulted in generation of a mutant virus that triggered muted cytokine activation in the spleen after i.v. administration [33]. Collectively, these data provide evidence that despite the multifaceted nature of innate immune activation and inflammatory response to adenovirus vectors, many critical steps of virus recognition by the innate immunity can be obviated through the introduction of structural modifications into adenovirus capsid proteins, thus allowing for the generation of adenovirus vectors with an improved safety profile.

The historical perception that adenovirus vectors are uniquely unsafe for therapeutic use in people undoubtedly stems from the tragic death of a patient, initially identified as subject 019 and later disclosed as Jesse Gelsinger, who was treated with a high dose of an adenovirus vector (3.8 × 10^13^ viral particles) that was administered via hepatic artery during a gene therapy trial in 1999 [13]. It is noteworthy that this same high dose of adenovirus vector was administered to a second participant in the trial, and that second patient experienced a significant but only transient elevation of IL-6 in the blood, while IL-6 continued to be highly elevated after virus administration to Jesse Gelsinger [13]. The mechanistic reason for this profoundly consequential divergence in cytokine response between two participants in this high dose vector cohort remains unknown. Nevertheless, a recent study analyzed the production of IL-6 and the expression of activation markers by in vitro differentiated dendritic cells after their exposure to adenovirus vector mixed with sera from healthy donors as well as with a frozen archived blood sample obtained from Jesse Gelsinger prior to adenovirus administration [87]. The authors found that exposure of dendritic cells to adenovirus vector mixed with blood of subject 019 triggered the production of very high amounts IL-6, which were significantly higher than the amounts of IL-6 released after cell exposure to adenovirus vector mixed with sera from 6 other donors tested in this study [87]. Although the authors suggested that pre-existing anti-HAdv-C5-specific antibodies may have played a role in potentiating virus entry into dendritic cells leading to potentiated IL-6 production, the amount of antibodies recognizing adenovirus proteins varied greatly between sera donors and were not in high amounts in the blood of subject 019, suggesting that a yet unidentified factor(s) present in the blood other than, or in addition to, virus-specific antibodies may be responsible for potentiating IL-6 production by the dendritic cells after their exposure to the adenovirus–serum mixture. Although limited by the number of serum samples analyzed, this study provides rationale for prospective analysis of serum samples from patients subject to therapy with adenovirus vectors to identify rare patients with virus hyper-sensitivity phenotype, like subject 019, that must be excluded from treatment cohorts or therapy with adenovirus vectors. Such a relatively simple, prospective patient stratification tool will prevent the administration of adenovirus to patients with inflammatory hypersensitivity response and thus reduce the risk of exuberant and potentially lethal inflammatory response to administration of the therapeutic vectors.

## 7. Summary

In summary, cytokines and chemokines are critical cell–cell communication proteins that become synthesized, activated, and released in a dose-dependent manner in response to natural adenovirus infection and in response to administration of therapeutic vectors based on adenovirus and other viral and non-viral delivery vectors [30,108,113,114,115]. Based on the abundance of accumulated data and improved understanding of specific signaling pathways that adenovirus triggers upon entry into immune phagocytic cells [30,56,57,58,59,60,61,62,63,64,65,66,67,68,69,70,71,72,73,74,75,76,77,78,79,116,117,118], one can conclude that the safety of adenovirus vectors can be managed through at least two independent and complementary approaches. The first approach of a “cytokine prophylaxis” involves administering currently available FDA-approved drugs that target key pro-inflammatory signaling pathways prior to, or together with, therapeutic vector administration. The second approach relies on the introduction of targeted mutations into adenovirus capsid to generate adenovirus vectors that avoid interaction with innate immune cells or cellular receptors that trigger exuberant cytokine responses. Furthermore, the insight obtained through analyzing dendritic cell responses to adenovirus mixture with serum samples in vitro provides the opportunity for the development of in vitro patient stratification tools for prospective identification of the rare patients with extreme inflammatory hypersensitivity phenotype. Such patients can therefore be excluded from recruitment to clinical trials or therapy cohorts, ensuring the safety of therapy for the patients in need. Future directions may focus on the identification of specific factors in the blood that trigger an inflammatory hypersensitivity response to adenovirus and optimization of clinical trial designs to allow for incorporation of all currently available approaches to improving safety of adenovirus-based therapeutics that aim to address the needs of patients that currently have limited or no therapeutic options.

## Data Availability

Not applicable.
